# Supplementary material for: Specific Cerebrospinal Fluid SerpinA1 Isoform Pattern in Alzheimer’s Disease
Source: Int J Mol Sci. 2022 Jun 22;23(13):6922. doi: 10.3390/ijms23136922 (PMC9266332; doi:10.3390/ijms23136922)
Supplement: Supplementary file 1 [file ijms-23-06922-s001.zip › ijms-1764367-supplementary.pdf]

International Journal of Molecular Sciences

MDPI

Supplementary Table S1. Correlation between AD biomarkers and serpinA1 isoform expression.

	Aβ42/40	t-tau	p-tau
	**All cohort**		
isoform 0	0.46 (0.31 to 0.59) ****	-0.37 (-0.51 to -0.21) ****	-0.34 (-0.48 to -0.17) ****
isoform 1	0.35 (0.19 to 0.50) ****	-0.33 (-0.48 to -0.16) ****	-0.31 (-0.46 to -0.15) ***
isoform 2	0.48 (0.34 to 0.61) ****	-0.50 (-0.62 to -0.36) ****	-0.50 (-0.62 to -0.36) ****
isoform 3	–	–	–
isoform 4	-0.36 (-0.51 to -0.20) ****	0.48 (0.32 to 0.59) ****	0.48 (0.33 to 0.60) ****
isoform 5	–	–	–
isoform 6	–	–	–
	**Patients with dementia (AD-dem, PDD or DLB)**		
isoform 0	–	-0.50 (-0.69 to -0.23) ***	-0.46 (-0.67 to -0.19) **
isoform 1	–	-0.55 (-0.73 to -0.30) ****	-0.62 (-0.78 to -0.40) ****
isoform 2	0.34 (0.04 to 0.58) *	-0.61 (-0.77 to -0.38) ****	-0.52 (-0.71 to -0.26) ***
isoform 3	–	–	–
isoform 4	–	0.66 (0.45 to 0.80) ****	0.68 (0.48 to 0.81) ****
isoform 5	–	–	–
isoform 6	–	–	–
	**Patients with MCI (AD-MCI or PD-MCI)**		
isoform 0	0.32 (0.02 to 0.56) *	–	-0.29 (-0.54 to -0.002) *
isoform 1	0.31 (0.01 to 0.55) *	–	–
isoform 2	0.42 (0.15 to 0.64) **	-0.47 (-0.67 to -0.21) ***	-0.51 (-0.70 to -0.26) ***
isoform 3	–	0.34 (0.05 to 0.57) *	0.39 (0.11 to 0.61) **
isoform 4	-0.45 (-0.66 to -0.18) **	0.30 (0.01 to 0.54) *	0.35 (0.07 to 0.58) *
isoform 5	–	–	–
isoform 6	–	–	–

Data are provided only when statistically significant and shown as Spearman r (95% confidence interval). *$p < 0.05$, **$p < 0.01$, ***$p < 0.001$, ****$p < 0.0001$. Abbreviations. Aβ42/40: amyloid-β_{1-42}/amyloid-β_{1-40} ratio; AD: Alzheimer's disease; AD-dem: Alzheimer's disease with dementia; AD-MCI: Alzheimer's disease with mild cognitive impairment; DLB: dementia with Lewy bodies; PDD: Parkinson's disease with dementia; PD-MCI: Parkinson's disease with mild cognitive impairment; p-tau: phosphorylated tau protein at threonine 181; t-tau: total tau protein.
